# Phenomena of hypo- and hyperconnectivity in basal ganglia-thalamo-cortical circuits linked to major depression: a 7T fMRI study

**DOI:** 10.1038/s41380-024-02669-4

**Published:** 2024-07-17

**Authors:** Jana Hagen, Shukti Ramkiran, Gereon J. Schnellbächer, Ravichandran Rajkumar, Maria Collee, Nibal Khudeish, Tanja Veselinović, N. Jon Shah, Irene Neuner

**Affiliations:** 1 Department of Psychiatry, Psychotherapy and Psychosomatics, Uniklinik RWTH Aachen, Aachen, Germany; 2 Institute of Neuroscience and Medicine - 4, Forschungszentrum Jülich, Jülich, Germany; 3 Department of Neurology, Uniklinik RWTH Aachen, Aachen, Germany; 4 Institute of Neuroscience and Medicine - 11, Forschungszentrum Jülich, Jülich, Germany

**Keywords:** Depression, Diagnostic markers

## Abstract

Major depressive disorder (MDD) typically manifests itself in depressed affect, anhedonia, low energy, and additional symptoms. Despite its high global prevalence, its pathophysiology still gives rise to questions. Current research places alterations in functional connectivity among MDD’s most promising biomarkers. However, given the heterogeneity of previous findings, the use of higher-resolution imaging techniques, like ultra-high field (UHF) fMRI (≥7 Tesla, 7T), may offer greater specificity in delineating fundamental impairments. In this study, 7T UHF fMRI scans were conducted on 31 MDD patients and 27 age-gender matched healthy controls to exploratorily contrast cerebral resting-state functional connectivity patterns between both groups. The CONN toolbox was used to generate functional network connectivity (FNC) analysis based on the region of interest (ROI)-to-ROI correlations in order to enable the identification of clusters of significantly different connections. Correction for multiple comparisons was implemented at the cluster level using a false discovery rate (FDR). The analysis revealed three significant clusters differentiating MDD patients and healthy controls. In Clusters 1 and 2, MDD patients exhibited between-network hypoconnectivity in basal ganglia-cortical pathways as well as hyperconnectivity in thalamo-cortical pathways, including several individual ROI-to-ROI connections. In Cluster 3, they showed increased occipital interhemispheric within-network connectivity. These findings suggest that alterations in basal ganglia-thalamo-cortical circuits play a substantial role in the pathophysiology of MDD. Furthermore, they indicate potential MDD-related deficits relating to a combination of perception (vision, audition, and somatosensation) as well as more complex functions, especially social-emotional processing, modulation, and regulation. It is anticipated that these findings might further inform more accurate clinical procedures for addressing MDD.

## Introduction

Major depressive disorder (MDD) refers to a condition characterized by symptoms such as depressed affect, anhedonia, low energy, aberrant appetite or sleep, feelings of insufficiency or guilt, concentration deficits, or suicidal ideas accumulating over a period of several weeks [1]. Those affected typically suffer great psychological stress and often struggle with social and occupational malfunctioning [2]. MDD is of universal relevance as it has one of the highest prevalences among psychiatric disorders worldwide [3].

Due to the complexity and phenotypic heterogeneity of MDD, its pathophysiology has yet to be fully elucidated. The current literature indicates closely intertwined mechanisms of abnormalities in neurotransmitter, stress, and immune systems, resulting in structural and functional alterations in the brain [4]. Thus, dysfunctional neuronal communication currently seems to be one of the most promising biomarkers of MDD. Respective investigations focus much on functional connectivity, which is defined as “the temporal correlation of a neurophysiological index measured in different brain areas” [5, p. 5] and typically assessed using functional magnetic resonance imaging (fMRI). However, in this respect, the findings of the previous literature are quite heterogeneous, not least in terms of methodology [6, 7].

The adoption of more advanced imaging techniques, specifically ultra-high field (UHF) MRI – with field strengths of 7 Tesla (7T) and above, may hold potential for mitigating the disparities in previous findings due to a significantly improved resolution [8]. The higher field strength offers key advantages, notably enhanced image signal-to-noise ratio (SNR) [9] and blood oxygen level-dependent (BOLD) contrast-to-noise ratio (CNR) [10, 11]. However, as the clinical availability of 7T UHF fMRI remains limited, it has rarely been used to investigate functional connectivity in MDD until now [12].

This study used 7T UHF fMRI to perform an exploratory analysis of functional connectivity patterns in MDD patients compared to healthy controls. The fMRI data were acquired during the resting state so that the mobilization of cognitive resources did not cause any interference. The analysis comprised the entire cerebrum and was conducted between all regions of interest (ROI) resulting from atlas-based segmentation. The goal of this approach was to delineate significant fundamental alterations in the functional connectivity of MDD patients, thus providing insights into the hitherto limited understanding of the disease’s pathophysiology.

Looking ahead, such studies might further help to explore the potential of UHF fMRI as a means to advance diagnosis, treatment, and response monitoring in psychiatry [13]. A more accurate clinical procedure is especially important in light of the “treatment-prevalence paradox”, which refers to the phenomenon that despite improved quality and accessibility of treatment, the prevalence of MDD persists. The reason for this does not appear to be a compensatory rise in cases but rather insufficient treatment benefits [14].

## Methods

### Participants

The study sample consisted of 31 MDD patients (age: 34.52 ± 12.48 years; gender: 16 females, 15 males) from the Department of Psychiatry, Psychotherapy and Psychosomatics of the Uniklinik RWTH Aachen and 27 healthy controls (age: 29.48 ± 10.04 years; gender: 11 females, 16 males) from the local community. A total sample size of 58 subjects was considered appropriate and in line with previous studies [6, 7, 12]. All patients met the ICD-10 and DSM-5 diagnostic criteria for MDD without psychotic symptoms. Their medical condition had to be sufficiently stable for the investigation. The health status of the control group was determined using the German version 6.0.0 of the Mini International Neuropsychiatric Interview (MINI) [15] to confirm the absence of mental disorders. Likewise, neurological disorders constituted an exclusion criterion for healthy controls. The groups were deemed to be matched in age and gender as they did not significantly differ in either variable (age: Mann-Whitney *U* = 298.50, *z* = −1.88, *p* = 0.061, range 18–63 years; gender: χ^2^(1) = 0.69, *p* = 0.408), calculated using IBM SPSS Statistics v29 software (IBM, Armonk, NY, USA). Further inclusion criteria comprised 7T UHF MRI compatibility and right-handedness, which was verbally assessed and, except for four subjects, additionally confirmed using a German version of the Edinburgh Handedness Inventory (EHI) [16]. All participants signed informed consent prior to the study, and the study was conducted in accordance with the Declaration of Helsinki and approved by the Ethics Committee of the Faculty of Medicine of the RWTH Aachen University.

### Data acquisition

The MRI data were recorded using a 7T MAGNETOM Terra scanner (Siemens Healthineers, Erlangen, Germany) located at the Forschungszentrum Jülich. It was operated with a 1Tx/32Rx-channel head coil (Nova Medical, Wilmington, MA, USA) for radiofrequency transmission and reception.

For the acquisition of the resting-state fMRI data, a 2D T2* weighted multiband accelerated echo planar imaging (EPI) protocol from the Center for Magnetic Resonance Research (CMRR, University of Minnesota, Minneapolis, MN, USA) was used [17]. It had a repetition time (TR) of 2000 ms, an echo time (TE) of 25 ms, and a flip angle (FA) of 70°, executed with a multiband factor of 4. A 1.3 mm isotropic resolution was obtained using a field of view (FOV) of 220 × 220 mm^2^, a matrix size of 168 × 168, and a slice thickness of 1.3 mm. In total, 305 volumes with 100 slices each were recorded. The acquisition lasted for approximately 10 min. Prior to onset, the participants were instructed to keep their eyes closed, to think of nothing specific and not to fall asleep. In addition, the lights were switched off. In order to enable the generation of fieldmaps needed for susceptibility distortion correction during preprocessing to counteract B_0_ field inhomogeneities, a “blip-up blip-down” acquisition was used [18]. For this purpose, the protocol was slightly modified by applying a 180° flip to the phase encoding direction, followed by the acquisition of two more volumes.

For the acquisition of the structural images, a 3D T1 weighted magnetization prepared two rapid acquisition gradient echoes (MP2RAGE) protocol was used. By applying different inversion times (TI) and FA, two inversion images (INV1 and INV2) were recorded and subsequently combined based on a ratio to achieve a structural image corrected for transmit and receive field bias as well as proton density and T2* contrast [19]. INV1 had a TI of 840 ms and a FA of 5°. INV2 had a TI of 2370 ms and a FA of 6°. The TR of 4500 ms and TE of 1.99 ms were the same for both images. A 0.75 mm isotropic resolution was obtained using a FOV of 225 × 240 mm^2^, a matrix size of 300 × 320, and a slice thickness of 0.75 mm. In total, 208 sagittal slices were acquired.

### Data preprocessing

The MRI data were converted from Digital Imaging and Communications in Medicine (DICOM) into Neuroimaging Informatics Technology Initiative (NIfTI) format using the tool “dcm2niix” included in the MRIcron v1.0.20190902 software (McCausland Center for Brain Imaging, University of South Carolina, Columbia, SC, USA). The images were then preprocessed, denoised, and analyzed using the functional connectivity toolbox CONN v21.a [20] based on SPM12 v7771 software (FIL Methods Group, University College London, London, UK) implemented in MATLAB R2021b v9.11 (The MathWorks, Natick, MA, USA). The fieldmaps required during preprocessing were precomputed using the tool “topup” [18] from FSL v6.0 software (FMRIB Analysis Group, University of Oxford, Oxford, UK).

The preprocessing was performed using one of the toolbox’s predefined pipelines called “preprocessing pipeline for volume-based analyses (direct normalization to MNI-space) when fieldmaps are available”. The applied parameters were kept in default settings. For the resting-state fMRI data, the preprocessing pipeline included the conversion of fieldmaps into voxel displacement maps, realignment and unwarping for motion and susceptibility distortion correction, translation of the image center into coordinates (0, 0, 0), slice timing correction for acquisition time differences, outlier detection based on artifact detection tools (ART) [21] with intermediate settings (97th percentiles in normative sample) to prepare scrubbing to eliminate volumes with exceeding motion or global BOLD signal, as well as segmentation into gray matter, white matter, and CSF and direct normalization into MNI152 standard space downsampled to a 2 mm isotropic resolution using a unified procedure [22]. For the structural images, the preprocessing pipeline included the translation of the image center into coordinates (0, 0, 0), as well as unified segmentation into gray matter, white matter, and CSF and normalization into MNI152 standard space downsampled to a 1 mm isotropic resolution.

Following preprocessing, denoising using linear nuisance regression was performed on the resting-state fMRI data in order to eliminate potential confounding influences from the BOLD signal. Again, default parameters were applied. The regressors consisted of five components each from white matter and CSF BOLD signals based on the anatomical component-based noise correction method (aCompCor) [23], six realignment parameters and their first derivatives, the scrubbing variables containing the outlier volumes, as well as factors for the session effect of resting state to offset initial transient BOLD signal instability and for linear detrending. Subsequently, temporal band-pass filtering at the default setting of 0.008–0.09 Hz was applied in order to additionally diminish noise, including that arising from physiological events like respiration and cardiac activity.

### Data analysis

For the first level analysis, the entire cerebrum was divided into 91 cortical and 14 subcortical parcellations using the default atlas offered by CONN, which is based on the 1 mm 25% thresholded Harvard-Oxford maximum likelihood atlas [24]. This resulted in a total of 105 ROIs. An ROI-to-ROI functional connectivity analysis was performed by setting up a hemodynamic response function (hrf) weighted general linear model (GLM) using bivariate correlations. These were calculated separately between each pair of ROIs so that a total of 5460 connections were considered. The resulting correlation coefficients were normalized using Fisher’s z-transformation.

In the second level analysis, the subject-specific ROI-to-ROI functional connectivity analysis was expanded by a group-level analysis, aiming to compare the correlation-based connectivity values in terms of differences between MDD patients and healthy controls. As it was of less interest to look at each individual connection between ROIs separately, but rather to infer from properly summarized groups of connections, the actual between-subjects group comparison was preceded by a data reduction step. A hierarchical clustering approach [25] was applied to sort individual ROIs into groups of ROIs (so-called “networks”). For this sorting, the complete linkage method of Euclidean distances was used, considering mainly functional connectivity (weighting criterion of 0.95) and to a lesser extent anatomical positional (weighting criterion of 0.05) similarities between ROIs. The total number of networks of ROIs was determined by the elbow method, locating the threshold where more networks of ROIs did not yield a significantly better explanation of the data. According to the default settings, the hierarchical clustering was performed within the whole combined sample of MDD patients and healthy controls, thus setting the framework for further analysis. This practice was kept in order to define a network model as the best possible representation of both subject groups. If the model had only been fit to one subsample, then the model would not have been fully appropriate for the other subsample in the subsequent group comparison. Further, due to the larger sample size, using the whole combined sample offered higher robustness for the network model definition.

Since the data reduction step enabled the conversion of the ROI-to-ROI analysis into a network-to-network analysis, a functional network connectivity (FNC) analysis [26] based on the definition of the between-subjects contrast [1 -1] was performed, constituting the core of the second level analysis. By means of a parametric multivariate pattern analysis (MVPA) omnibus test relying on a GLM using F-tests, a comparison of the FNC of MDD patients and healthy controls was calculated separately for each possible group of all individual ROI-to-ROI connections comprised within each predefined network and between each pair of networks of ROIs (so-called “clusters”). A correction for multiple comparisons was realized at the cluster level by adjusting their p-values for a false discovery rate (FDR) [27]. Thus, clusters with an FDR-corrected *p* < 0.05 were considered significantly different between MDD patients and healthy controls. In addition, post-hoc two-sided t-tests using a threshold of uncorrected *p* < 0.05 were performed to identify those individual ROI-to-ROI connections that contributed to each cluster of significant between-subjects group differences. The subsequent representation of the composition of clusters providing significant results focuses on their post-hoc identified significantly involved components.

The description of CONN’s preprocessing and analysis steps follows the user interface and handbook of the toolbox [28].

## Results

**Fig. 1:**
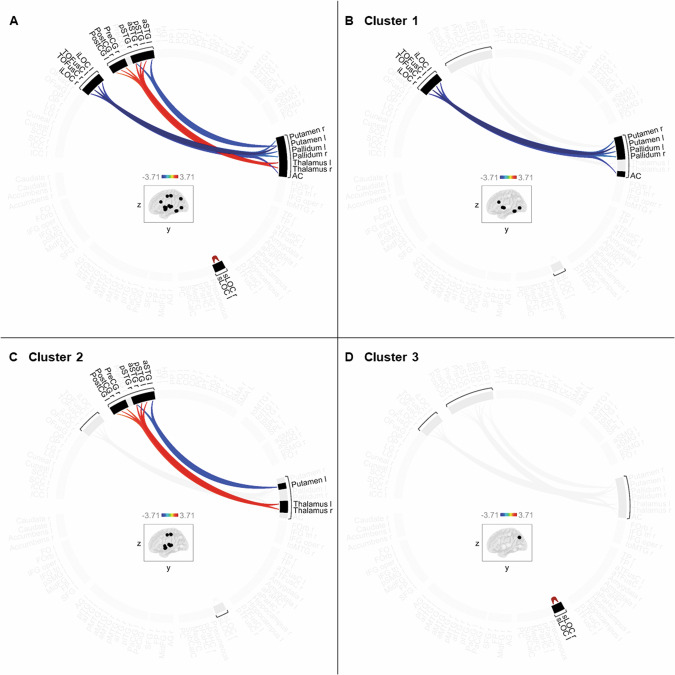
Resting-state functional connectivity clusters of significant differences between MDD patients and healthy controls. Depicted collectively (**A**) and separately (**B**–**D**). Contrast [1 -1]: MDD patients > healthy controls. Blue: decreased connectivity, red: increased connectivity. aSTG: anterior superior temporal gyrus, pSTG: posterior superior temporal gyrus, PreCG: precentral gyrus, PostCG: postcentral gyrus, iLOC: inferior lateral occipital cortex, TOFusC: temporal-occipital fusiform cortex, AC: anterior cingulate gyrus, sLOC: superior lateral occipital cortex; l: left, r: right.

The resting-state FNC analysis across the entire cerebrum of MDD patients and healthy controls revealed among 136 possible within-network and between-network clusters three clusters of significant differences between both groups (Fig. 1A).

### Cluster 1

**Fig. 2:**
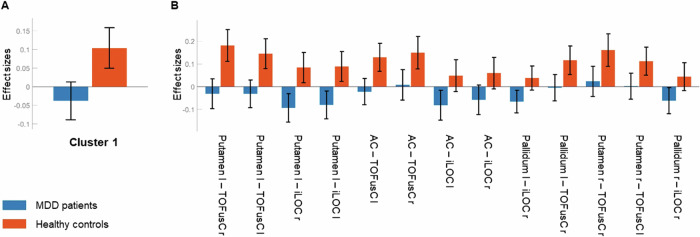
Cluster 1: Mean connectivity values and 90% confidence intervals (Fisher’s z-transformed correlation coefficients) within each group. Averaged (**A**) and ROI-to-ROI specific (**B**). Blue: MDD patients, red: healthy controls. TOFusC: temporal-occipital fusiform cortex, iLOC: inferior lateral occipital cortex, AC: anterior cingulate gyrus; l: left, r: right.

Cluster 1 (Fig. 1B) displayed decreased between-network connectivity in MDD patients compared to healthy controls, *F*(2, 55) = 14.77, *p*_*FDR*_ < 0.001. In one network, the bilateral putamen, bilateral pallidum, and anterior cingulate gyrus (AC) were involved, and the other network comprised the bilateral temporal-occipital fusiform cortex (TOFusC) and bilateral inferior lateral occipital cortex (iLOC). Post-hoc t-tests of individual ROI-to-ROI connections are listed in Table 1A. As indicated by post-hoc plots (Fig. 2), the decreased connectivity resulted from MDD patients exhibiting no or rather slightly negative (i.e. anticorrelated) connectivity and healthy controls exhibiting rather slightly positive connectivity across the connections in Cluster 1.

**Table 1:** Post-hoc t-tests of individual ROI-to-ROI connections within each cluster (A–C).

Individual connection	t-statistic, *df* = 56	*p*_*uncorrected*_
A Cluster 1		
Putamen l – TOFusC r	−3.71	<0.001
Putamen l – TOFusC l	−3.31	0.002
Putamen l – iLOC r	−3.25	0.002
Putamen l – iLOC l	−3.16	0.003
AC – TOFusC l	−2.99	0.004
AC – TOFusC r	−2.42	0.019
AC – iLOC l	−2.26	0.028
AC – iLOC r	−2.07	0.043
Pallidum l – iLOC r	−2.39	0.020
Pallidum l – TOFusC r	−2.36	0.022
Putamen r – TOFusC r	−2.35	0.023
Putamen r – TOFusC l	−2.19	0.033
Pallidum r – iLOC r	−2.10	0.040
B Cluster 2		
Putamen l – aSTG l	−2.71	0.009
Putamen l – pSTG r	−2.36	0.022
Thalamus l – aSTG r	3.18	0.002
Thalamus l – pSTG l	2.86	0.006
Thalamus r – aSTG r	2.57	0.013
Thalamus r – PostCG r	2.54	0.014
Thalamus r – pSTG r	2.38	0.021
Thalamus r – pSTG l	2.34	0.023
Thalamus r – PreCG r	2.25	0.028
Thalamus r – PostCG l	2.19	0.032
Thalamus l – pSTG r	2.60	0.012
Thalamus l – PreCG r	2.27	0.027
Thalamus l – PostCG r	2.18	0.034
C Cluster 3		
sLOC l – sLOC r	3.59	<0.001

Contrast [1 -1]: MDD patients > healthy controls.

*df* degrees of freedom, *p*_*uncorrected*_ significance threshold of uncorrected *p* < 0.05, *aSTG* anterior superior temporal gyrus, *pSTG* posterior superior temporal gyrus, *PreCG* precentral gyrus, *PostCG* postcentral gyrus, *iLOC* inferior lateral occipital cortex, *TOFusC* temporal-occipital fusiform cortex, *AC* anterior cingulate gyrus, *sLOC* superior lateral occipital cortex, *l* left, *r* right.

### Cluster 2

**Fig. 3:**
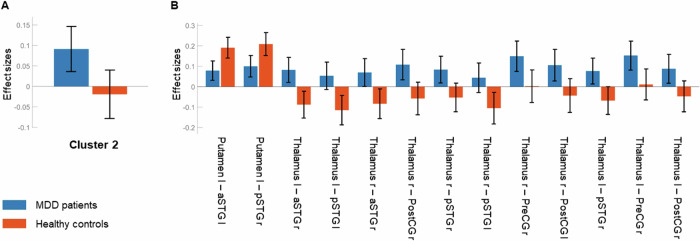
Cluster 2: Mean connectivity values and 90% confidence intervals (Fisher’s z-transformed correlation coefficients) within each group. Averaged (**A**) and ROI-to-ROI specific (**B**). Blue: MDD patients, red: healthy controls. aSTG: anterior superior temporal gyrus, pSTG: posterior superior temporal gyrus, PostCG: postcentral gyrus, PreCG: precentral gyrus; l: left, r: right.

In Cluster 2 (Fig. 1C), MDD patients showed decreased as well as increased between-network connectivity compared to healthy controls, *F*(2, 55) = 9.88, *p*_*FDR*_ = 0.015. Following the logic of the analysis, both connectivity patterns were based on the same networks. The decreased connectivity occurred between the left putamen and the left anterior and right posterior superior temporal gyrus (aSTG and pSTG). The increased connectivity emerged between the bilateral thalamus and the right aSTG, bilateral pSTG, right precentral gyrus (PreCG), and bilateral postcentral gyrus (PostCG). For post-hoc t-tests of individual ROI-to-ROI connections, see Table 1B. Post-hoc plots (Fig. 3) show that MDD patients exhibited rather slightly positive connectivity across the connections in Cluster 2. The decreased connectivity in the connections involving the left putamen arose from healthy controls exhibiting more slightly positive connectivity than MDD patients. The increased connectivity in the connections involving the bilateral thalamus resulted from healthy controls exhibiting no or rather slightly negative (i.e. anticorrelated) connectivity.

### Cluster 3

**Fig. 4:**
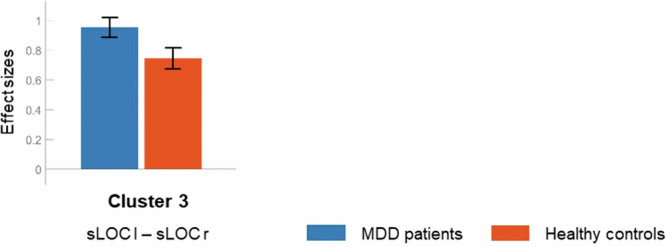
Cluster 3: Mean connectivity values and 90% confidence intervals (Fisher’s z-transformed correlation coefficients) within each group. Blue: MDD patients, red: healthy controls. sLOC: superior lateral occipital cortex; l: left, r: right.

Cluster 3 (Fig. 1D) demonstrated increased within-network connectivity in MDD patients compared to healthy controls, *F*(1, 56) = 12.88, *p*_*FDR*_ = 0.032. The network comprised the left and right superior lateral occipital cortex (sLOC). Despite only containing one individual connection, the post-hoc t-test is shown in Table 1C for completeness. According to the post-hoc plot (Fig. 4), both groups exhibited significant positive connectivity in Cluster 3, with MDD patients exceeding healthy controls.

## Discussion

The exploratory analysis of 7T UHF resting-state fMRI data of the entire cerebrum in MDD patients compared to healthy controls identified significant disease-related functional connectivity alterations. Hypo- and hyperconnectivity were evident in three separate clusters, including several individual ROI-to-ROI connections. Clusters 1 and 2 suggest associations with pathways of the basal ganglia-thalamo-cortical circuits, and Cluster 3, with its specificity for the interhemispheric connectivity of visual areas, provides additional evidence of alterations in perception and more complex functions in MDD patients.

The basal ganglia refer to a subcortical formation, which mainly includes the caudate nucleus, putamen, and pallidum [29], and have been found to be significant in MDD-related altered functional connectivity [6]. By receiving input from various cortical regions and sending output back to the cortex, primarily the frontal lobe, the basal ganglia form circuits that are well-known for their motor functions [30, 31]. Within these circuits, the thalamus plays an important role. By acting as both a relay and filter station, the thalamus influences to varying degrees the information sent to the cortex and thus its further conscious processing [32, 33]. Previously, five basal ganglia-thalamo-cortical circuits along with modulatory subcircuits have been suggested to be associated with distinguishable cortical functions [34]. More recent findings point to the basal ganglia, thalamus, and various cortical regions as components of an intricate, highly interconnected network system that serves multiple motor, cognitive, and emotional functions [35, 36].

### Cluster 1

Cluster 1 is characterized by MDD-related hypoconnectivity in both basal ganglia-cortical and cortico-cortical pathways.

Specifically, the cortical ROIs involved in the basal ganglia-cortical pathways, TOFusC and iLOC, belong to the associative visual cortex. They are partly made up of two of three core systems of the face perception network: the fusiform face area (FFA) in the TOFusC and the occipital face area (OFA) in the iLOC [37]. Both regions are specialized in face recognition. The OFA is assumed to operate at an early processing level, registering physical characteristics such as facial elements [38]. The FFA is considered to be guided by more elaborate stable attributes representing the distinctive identity of faces [39]. MDD patients showing decreased connectivity between the putamen and pallidum as important basal ganglia input and output structures and the TOFusC and iLOC suggest a disturbed modulation of facial information. These findings are supported by previous studies demonstrating the involvement of the respective ROIs in MDD-related hypoconnectivity patterns. With regard to the putamen and pallidum, a broad subcortical cluster was shown to exhibit decreased connectivity in MDD patients compared to healthy controls [40]. Further, decreased connectivity between a cluster, mainly composed of the temporal-occipital fusiform gyrus and lateral occipital cortex, and the posterior cingulate cortex (PCC) was observed to be associated with increased suicidality in adolescent MDD patients [41].

Face recognition serves as a basic requirement that influences important subsequent functions, such as emotion recognition. Evidence exists that particularly the FFA is also susceptible to emotional expressions [42, 43]. The alterations found in Cluster 1 could potentially be associated with deficits in face and emotion recognition, and thus are in line with the hypothesis of mood-congruent biased memory and thinking [44] as well as with the “cognitive triad” of negatively biased thoughts about the self, the world and the future in MDD patients [45]. Both models suggest a significant impact on perceptual processes, which may extend to facial emotion recognition, as MDD patients seem to be less able to accurately recognize most basic emotions, including anger, disgust, fear, and happiness. However, this does not appear to apply to sadness, where reactivity might be elevated [46]. Such alterations in facial emotion processing are indeed linked to structures emerging in Cluster 1. There is evidence that the association between the intensity of facial emotions and the response in the fusiform gyrus and putamen is negative for happiness and positive for sadness in MDD patients, whereas the opposite was observed in healthy controls [47]. In addition, MDD patients typically show a reduced capacity for emotional modulation, especially with regard to positive affect [48]. Intriguingly, the phenotype of reduced facial modulation can also be found in neurodegenerative disorders of subcortical structures, which include the basal ganglia, such as in Parkinson’s disease (PD) [49]. Fittingly, hypomimia in PD is also assumed to be associated with impairments in facial emotion recognition [50]. In this context, MDD patients often suffer from interpersonal difficulties due to compromised social competence [51].

The aforementioned inferences from Cluster 1 become even more substantial considering the cortico-cortical pathways involving the AC. The AC partly encloses the basal ganglia and belongs to the limbic system, which is well-known for its role in emotional processing [52]. By acting at the transition between emotional and cognitive functions, the AC is specialized in emotion regulation [53]. Decreased connectivity between the AC and the TOFusC and iLOC in MDD patients suggests that, in addition to the modulation of facial and emotional information in the basal ganglia, its monitoring and control might also be disturbed in MDD patients. This constellation implies a self-reinforcing process.

### Cluster 2

Cluster 2 is characterized by MDD-related hypoconnectivity in basal ganglia-cortical pathways and hyperconnectivity in thalamo-cortical pathways.

The cortical ROIs involved in the hypoconnectivity pattern, aSTG and pSTG, can be assumed to contain activation originating from the superior temporal sulcus (STS), as the Harvard-Oxford atlas does not label any sulci. The STS is of special interest as it represents the third core system, besides FFA and OFA, of the face perception network, which is specialized in processing variable features of faces [37]. The posterior STS (pSTS) is most reliably found to be involved in face and especially emotion recognition, but the system may also extend to the anterior STS (aSTS) [54]. A UHF fMRI study supports this view by showing that varying facial stimuli, including emotional expressions as well as lip and eye movements, evoke activation in intersecting but separable regions along the STS [55]. MDD patients showing decreased connectivity between the putamen and the aSTG and pSTG again suggest dysfunctional modulation of emotional and other facial expressions in the basal ganglia. Consistent with these findings, similar decreased connectivity between the putamen and the STG was previously shown in MDD patients compared to healthy controls [56]. In addition to Cluster 1, MDD-related alterations in facial emotion processing are also associated with structures emerging in Cluster 2. After congruent mood induction, decreased activation in the posterior temporal lobe in response to happy facial stimuli and increased activation in the transverse temporal gyrus adjacent to the STG in response to sad facial stimuli was found in MDD patients compared to healthy controls [57]. This further indication of deficient decoding of emotional and other facial expressions renders difficulties in social situations in MDD patients even more plausible, as they might miss or misinterpret nonverbal messages that are provided in this way [58].

The most well-known function of the STG is hosting the auditory cortex [59]. Similar to facial emotion expressions, the processing of auditory emotional information, especially from the human voice, is also located in this region [60, 61]. The significant role of voice, regardless of whether it is speech or not, is reflected by the existence of a specialized temporal voice area (TVA) along the STS and STG [62]. Intersecting with the TVA, a further subregion exists, with maximal activation in the middle STG, corresponding to the emotional voice area (EVA), which is specifically susceptible to voices containing emotional expressions [63]. Given this context, the MDD-related hypoconnectivity between the putamen and the aSTG and pSTG seen in Cluster 2 further suggests deficits in the modulation of auditory, especially vocal emotional information. This again emphasizes the interpersonal relevance of the found alterations in MDD patients. Potentially impaired emotion decoding in two sensory modalities simultaneously is likely to cause cumulative difficulties in social situations. Indeed, there is evidence of MDD patients being less able to accurately recognize emotions from auditory stimuli. It was shown that MDD patients exhibited increased miscategorization of neutrality and happiness in piano music as well as of neutrality and surprise in non-speech voices, whereas recognition of respective other emotions, including sadness and fear, was preserved. In line with a negativity bias, they overly mistook neutrality for negative emotions in the vocal condition and mostly experienced those with greater intensity than healthy controls [64]. There is also evidence that auditory emotion recognition is broadly compromised in children suffering from depressive symptoms [65].

The cortical ROIs involved in the hyperconnectivity pattern besides aSTG and pSTG, PreCG and PostCG, comprise the sensorimotor cortex. The PreCG hosts the primary motor cortex, and the PostCG hosts the primary somatosensory cortex, separated by the central sulcus where the frontal lobe merges into the parietal lobe [66]. MDD patients showing increased connectivity between the thalamus as relay and filter station in basal ganglia-thalamo-cortical circuits and the aSTG, pSTG, PreCG, and PostCG suggest an informational dysregulation both in the auditory system, including the processing of salient and emotional expressions from faces and voices, as well as in the sensorimotor system. Consistent with these findings, similar increased connectivity between the thalamus and the temporal and somatosensory cortex was previously demonstrated in MDD patients compared to healthy controls [67]. Intriguingly, thalamic hyperconnectivity was shown to be crucial for differentiating MDD patients and healthy controls, although the majority of the MDD patients’ brain was characterized by decreased connectivity [68]. The latter fits well with the hypoconnectivity patterns in Clusters 1 and 2 discussed before.

Regarding the aSTG and pSTG, supposing that MDD patients experience sensory overload beyond the aforementioned functions, it is noteworthy that the STG and STS play a role in inner verbalization phenomena like covert speech, verbal imagery, and silent reading [69]. Similarly, rumination, a common symptom of MDD, primarily occurs as inner verbalization [70, 71]. In doing so, MDD patients are involved in repetitive self-oriented negative thinking, a dysfunctional emotion regulation strategy preventing the processing of negative experiences by constant re-evaluation [72]. Indeed, there is evidence of rumination induction in healthy subjects leading to activation in the STG [73].

In terms of the PreCG and PostCG, somatic symptoms accompanying MDD are of special interest. In particular, psychomotor agitation stands out in the context of the found alterations potentially associated with deficits in sensorimotor regulation. It typically manifests itself in both physical restlessness as well as inner tension [74]. Psychomotor agitation can be characterized by increased connectivity between the thalamus and the sensorimotor network (SMN) [75], similar to parts of the hyperconnectivity pattern in Cluster 2. Moreover, MDD is associated with the tendency to perceive inexplicable physical discomfort, which resembles somatization disorder (SD), a common comorbidity of MDD [76]. There is evidence that SD patients also differ from healthy controls by thalamic hyperconnectivity involving the PreCG and PostCG [77]. A similar condition of increased somatosensory sensitivity associated with MDD refers to the intensified perception of pain [78]. In line with the aforementioned considerations, higher experienced pain intensity and increased activation in the PostCG were found in MDD patients with pain than in MDD patients without pain during pain induction [79]. Furthermore, the PreCG and PostCG have been linked to emotional processing, especially empathy, an important interpersonal function [80, 81]. Fittingly, there is evidence that watching pain in others evokes responses in sensorimotor areas [82]. In MDD patients, empathy is associated with difficulties in distancing oneself from the pain experienced by others, making them feel responsible and guilty [83]. This view expands the found alterations by potential additional MDD-related deficits in social-emotional regulation.

### Cluster 3

Cluster 3 differs from Clusters 1 and 2 for several reasons. Instead of containing multiple ROIs, it relies on only two, the left and right sLOC. Cluster 3 represents a cortico-cortical pathway, unlike the majority of subcortico-cortical pathways previously discussed. Thus, Cluster 3 is not part of the basal ganglia-thalamo-cortical circuits in the strict sense. Moreover, Cluster 3 has noticeably higher absolute connectivity values in both groups, with hyperconnectivity in MDD patients compared to healthy controls.

Interhemispheric connectivity is commonly analyzed using voxel-mirrored homotopic connectivity (VMHC), a temporal correlation measure of the BOLD signal in matching bilateral voxel pairs [84]. Supporting the findings in Cluster 3, VMHC-based increased interhemispheric connectivity in the superior and middle occipital gyrus was demonstrated to differentiate MDD patients and healthy controls [85]. In addition to Cluster 1, the sLOC represents another part of the visual cortex that appears to be impaired in MDD patients. This renders it plausible that the sLOC, beyond its purely visual function, might also be linked to social-emotional processing. Indeed, increased sLOC activation in response to neutral compared to happy and sad videos was found in healthy subjects [86]. In this sense, sLOC hyperconnectivity may reflect a general state of flat affect in MDD patients. Furthermore, there is evidence that the accumulation of stressful life events has a neuronal modulatory effect during emotion regulation. It was shown that activation in the sLOC and adjacent superior parietal lobule (SPL) increased while regulating exposure to negative pictures and decreased while regulating exposure to positive pictures in subjects with greater stress, whereas the opposite was observed in subjects with less stress [87]. As MDD is associated with different types of stress [88], the sLOC hyperconnectivity may also reflect an enhanced involvement in regulating negative thoughts. This view fits with the aforementioned MDD-related concepts of negativity bias and rumination.

As the ROI corresponding to the sLOC defined by the Harvard-Oxford atlas is spatially extensive, it can be assumed that it also contains activation originating from networks with a posterior parietal focus, such as the default mode network (DMN) or the dorsal attention network (DAN) [89, 90]. The DMN refers to the brain’s basic activation in the absence of stimulation [91], whereas the DAN is responsible for the top-down allocation of attention [92]. Although the use of VMHC mostly shows MDD-related posterior parietal interhemispheric hypoconnectivity, particularly in the DMN [93], an approach that adjusts for intrahemispheric connectivity offers a different perspective. Accordingly, posterior parietal regions, like the PCC and precuneus of the DMN as well as the SPL and intraparietal sulcus (IPS) of the DAN, were found to exhibit a greater tendency to work rather inter- than intrahemispherically in MDD patients compared to healthy controls [94].

Consideration should also be given to the DMN and DAN in the interpretation of Cluster 3, as there is evidence of increased connectivity between the DMN and DAN in MDD patients compared to healthy controls [95]. Moreover, persistent stressful circumstances were demonstrated to be linked to elevated depressive symptoms as well as increased connectivity in posterior parietal regions of both the DMN and DAN in healthy subjects [96]. With particular regard to the posterior parietal DMN, general hyperactivation and hyperconnectivity are associated with MDD [97]. Taken together, these findings support those in Cluster 3 and point to a dysregulated network implementation in MDD patients. Therefore, the potential involvement of the DMN, especially, and DAN in the hyperconnectivity between the left and right sLOC suggests an MDD-related basally enhanced internal focus. Finally, the observed higher absolute connectivity values in both groups become plausible in light of the involvement of the DMN, the most prominent resting-state network. Also, VMHC-based interhemispheric connectivity is considered inherently pronounced, not least in the parieto-occipital cortex [84, 93].

Looking at all clusters together reveals that most regions in the cortical networks (TOFusC, iLOC, aSTG, pSTG, PreCG, PostCG, and sLOC) are parts of the sensory pathways for vision, audition, and somatosensation. At first glance, this indicates a focus on potential MDD-related deficits in perception. Nevertheless, roles beyond pure perceptual functions are suggested, pointing to impairments in social-emotional processing, modulation, and regulation. Since all structures in the predominantly subcortical network (putamen, pallidum, thalamus, and AC) belong to the reward system [98], impaired reward processing also seems to play a role.

Technically, the benefit of the study resulting from the application of 7T UHF fMRI is based on the key advantages mentioned before, namely that the higher field strength provides increased resolution in terms of greater SNR and CNR [8–11]. With regard to the specific use of ROI-to-ROI-based FNC analysis, it can be expected that with higher resolution, more information in the form of voxels as measurement units of activation is present within each ROI. This means that 7T UHF fMRI offers the possibility to detect ROI-related signal that may not be measurable at lower field strengths, thus enabling the identification of more subtle effects.

## Conclusions

The use of 7T UHF fMRI analysis to explore fundamental MDD-related alterations in cerebral resting-state functional connectivity patterns provides valuable insights into the pathophysiology of the disease, particularly regarding the functionality of basal ganglia-thalamo-cortical circuits. The inferences about the vulnerability of these circuits and consequences when affected might further expand the clinical understanding of MDD, offering the potential to improve diagnosis, treatment, and response monitoring.

### Limitations

The findings of this study may be limited by differences in the psychopharmaceutical treatment and in the prevalence of comorbid disorders among MDD patients. At the same time, obtaining the effects regardless of somewhat variable conditions stresses the generality of the findings. Nevertheless, the findings of this study remain exploratory, as the sample used was limited in size. In order to assess the reliability of the observed effects, future investigations with larger data sets are needed.

## Data Availability

Data will be made available on request.
